# Predictors of competitive employment in individuals with severe mental illness: results from an observational, cross-sectional study in Germany

**DOI:** 10.1186/s12995-022-00345-3

**Published:** 2022-01-18

**Authors:** Uta Gühne, Alexander Pabst, Markus Kösters, Alkomiet Hasan, Peter Falkai, Reinhold Kilian, Andreas Allgöwer, Klemens Ajayi, Jessica Baumgärtner, Peter Brieger, Karel Frasch, Stephan Heres, Markus Jäger, Andreas Küthmann, Albert Putzhammer, Bertram Schneeweiß, Michael Schwarz, Thomas Becker, Johanna Breilmann, Steffi G. Riedel-Heller

**Affiliations:** 1 Institute of Social Medicine, Occupational Health and Public Health (ISAP), Medical Faculty, University of Leipzig, Philipp-Rosenthal-Straße 55, 04103 Leipzig, Germany; 2 Department of Psychiatry and Psychotherapy II, Ulm University, BKH Günzburg, Günzburg, Germany; 3 Department of Psychiatry, Psychotherapy and Psychosomatic, University of Augsburg, Medical Faculty, BKH Augsburg, Augsburg, Germany; 4 Department of Psychiatry and Psychotherapy, University Hospital Munich, Munich, Germany; 5 Institute for Epidemiology and Medical Biometry, Ulm University, Ulm, Germany; 6 Kbo-Isar-Amper Hospital, Region Munich, Germany; 7 Department of Psychiatry, Psychotherapy and Psychosomatic, District hospital Donauwörth, Donauwörth, Germany; 8 Department of Psychiatry, Psychotherapy and Psychosomatic, District hospital Kempten, Kempten, Germany; 9 Department of Psychiatry, Psychotherapy and Psychosomatic, District hospital Memmingen, Memmingen, Germany; 10 Department of Psychiatry, Psychotherapy and Psychosomatic, District hospital Kaufbeuren, Kaufbeuren, Germany

**Keywords:** Severe mental illness, Employment, Supported employment, Predictors, Physical health, Psychosocial functioning

## Abstract

**Background:**

Employment is of great importance as it is associated with various positive effects. Individuals with severe mental illness (SMI) are often excluded from competitive employment. Current data on employment of individuals with mental illness are rare, and influencing factors are under-researched. The present study examines possible predictors of competitive employment among individuals with SMI.

**Methods:**

This was a cross-sectional and multicentered study of 300 individuals with SMI aged 18 to 65 years. The following inclusion criteria were used: (I) diagnosis of schizophrenia, schizotypal and delusional disorders (ICD-10 F2x), or affective disorders (ICD-10 F3x), (II) duration of psychiatric illness ≥ 2 years, and (III) substantial impact of illness on social functioning. Participants were interviewed by trained staff using standardised instruments. The relationship between potential predictors (age, sex, education, marital status, living situation, migration background, psychosocial functioning, age at first mental problem, physical illness, work ability) and employment was analysed using a hierarchic binary logistic regression model.

**Results:**

Only one-third (34%) of participants were competitively employed. Almost one-third were unemployed (30%), and 28% reported early retirement due to mental illness. Psychosocial functioning was positively associated with competitive employment (OR = 1.09, 95% CI: 1.05 – 1.13, *p *< 0.001); concurrent chronic physical illness was negatively associated with competitive employment (OR = 0.38, 95% CI: 0.21 – 0.71, *p* = 0.002).

**Conclusions:**

Findings confirm a high risk of exclusion from competitive employment among individuals with SMI. Nonetheless, a substantial proportion of individuals are employed. Findings call for efforts to maintain or enhance workforce participation among individuals with SMI. A special focus should be placed on improving physical health and strengthening psychosocial functioning.

**Trial registration:**

The study was registered in the German Clinical Trials Register (DRKS) under the registration number DRKS00015801 before the start of recruitment (Registration date: 21.02.2019).

## German abstract

**Hintergrund:**

Berufliche Beschäftigung ist von großer Bedeutung für den Einzelnen, da sie mit verschiedenen positiven Effekten verbunden ist. Menschen mit schweren psychischen Erkrankungen sind oft vom allgemeinen Arbeitsmarkt ausgeschlossen. Aktuelle Daten zur Beschäftigung von Menschen mit psychischen Erkrankungen liegen kaum vor und die Einflussfaktoren sind bisher wenig erforscht. Die vorliegende Studie untersucht mögliche Prädiktoren für eine wettbewerbsfähige Beschäftigung bei Menschen mit schweren psychischen Erkrankungen.

**Methoden:**

Berichtet werden Ergebnisse einer multizentrischen Querschnittsstudie mit 300 Menschen mit schweren psychischen Erkrankungen im Alter von 18 bis 65 Jahren. Die folgenden Einschlusskriterien wurden verwendet: (I) Diagnose einer Schizophrenie, schizotypen oder wahnhaften Störung (ICD-10 F2x) oder affektiven Störung (ICD-10 F3x), (II) Dauer der psychiatrischen Erkrankung ≥ 2 Jahre und (III) erhebliche Auswirkungen der Erkrankung auf das soziale Funktionsniveau. Die Teilnehmenden wurden von geschultem Personal mit standardisierten Instrumenten befragt. Der Zusammenhang zwischen möglichen Prädiktoren (Alter, Geschlecht, Bildung, Familienstand, Wohnsituation, Migrationshintergrund, psychosoziale Funktionsfähigkeit, Alter bei ersten psychischen Problemen, körperliche Erkrankung, Arbeitsfähigkeit) und der Beschäftigung wurde mittels eines hierarchischen binären logistischen Regressionsmodells analysiert.

**Ergebnisse:**

Nur ein Drittel (34 %) der Teilnehmenden war kompetitiv beschäftigt. Fast ein Drittel der Teilnehmenden war arbeitslos (30 %), und 28 % berichteten über eine Frühverrentung aufgrund der psychischen Erkrankung. Die psychosoziale Funktionsfähigkeit war positiv mit kompetitiver Beschäftigung assoziiert (OR = 1,09, 95% CI: 1,05 - 1,13, *p* < 0,001); gleichzeitige chronische körperliche Erkrankungen waren negativ mit kompetitiver Beschäftigung assoziiert (OR = 0,38, 95% CI: 0,21 - 0,71, *p *= 0,002).

**Schlussfolgerungen:**

Die Ergebnisse bestätigen ein hohes Risiko des Ausschlusses von kompetitiver Beschäftigung bei Menschen mit schwerer psychischer Erkrankung. Gleichwohl ist ein beträchtlicher Anteil beschäftigt. Die Bemühungen, die Erwerbsbeteiligung der Betroffenen zu erhalten bzw. zu erweitern, müssen intensiviert werden. Ein besonderer Fokus sollte dabei auf die körperliche Gesundheit sowie die Stärkung der psychosozialen Funktionen gelegt werden.

## Background

Work and unemployment have an important impact on health and well-being [1, 2]. This applies to individuals without and with mental illness. As early as 1933, Jahoda et al. were able to show that gainful employment not only had the function of earning money for individuals, but also fulfilled other functions. Accordingly, it provides social contacts and a time structure, gives impulses for activity and conveys social status [3].

Positive effects of work on numerous outcomes, such as psychopathological symptoms [4–6], psychosocial functions [7, 8], self-esteem [4, 5, 9] and quality of life [10, 11], higher satisfaction with the financial situation [4, 5] as well as a reduced use of psychiatric inpatient [6, 10] and outpatient treatment [9] could also be shown for people with severe mental illness (SMI). In comparison to other forms of work and employment, a unique position of competitive employment is emerging [4–6, 8]. A systematic review examining the influence of employment on the course of severe mental illness also showed that taking up employment does not negatively influence the course of the illness [9]. According to this, accessing and maintaining employment is a high priority for individuals with SMI, which has apparently been given too little consideration in mental health care so far.

With increasing mental illness severity, the employment rate decreases [12]. Internationally, employment rates for individuals with a diagnosis of schizophrenia are estimated at 10 to 20% [13]. A more recent study of individuals undergoing inpatient psychiatric treatment in Germany showed that only about 20% of theme had a permanent employment contract [14]. Furthermore, authors report that a substantial proportion of individuals with SMI failed to return to their workplace after discharge. The link between mental health and work and the driving forces behind work outcomes of individuals with SMI are still too little known [15].

This study aims to describe the association of characteristics of individuals with SMI with competitive employment.

The following questions will be investigated:

1. How many individuals with SMI are currently competitively employed?
2. What distinguished individuals with SMI who are competitively employed from those who are not? What are the determining sociodemographic, illness-related and other factors?

## Methods

### Design and Setting

This study was a non-interventional, cross-sectional study of individuals with SMI conducted within a larger project (Implementation Status of the German Guideline for Psychosocial Interventions for Patients with Severe Mental Illness; IMPPETUS) [16]. The ethical approval was obtained from the ethics committee of Ulm University (ref: 463/18). Participants were recruited in ten psychiatry and psychotherapy departments that provide in- and outpatient psychiatric care for individuals with mental illnesses in Bavaria (Upper Bavaria and Swabia), including metropolitan catchment areas (Augsburg, Munich), middle-urban regions (Kempten, Memmingen) and rural regions (Donauwoerth, Guenzburg, Kaufbeuren, Taufkirchen). Recruitment and data collection were carried out from March 2019 to September 2019. Study participants were informed about the study via an information sheet and were asked to provide written informed consent to participate. They were interviewed during their inpatient or day-hospital stay.

### Inclusion criteria

To identify individuals with SMI, the following inclusion criteria were used: (I) diagnosis of schizophrenia, schizotypal and delusional disorders (ICD-10 F2x) or affective disorders (ICD-10 F3x), (II) duration of psychiatric illness ≥ 2 years, and (III) substantial consequences for activities of daily life and social functioning [17]. The following thresholds were applied to operationalize (III): 1) GAF [18] from ≤ 60, and 2) HoNOS [19] score of (a) ≥ 2 on one of the items of the symptomatic problems subscale (scores 6, 7 and 8) and a score of ≥ 2 on each of the four items of the social problems subscale (scores 9, 10, 11 and 12), or (b) a score of ≥ 3 on at least one of these items (9, 10, 11 or 12). The HoNOS-D is a 12-item instrument for recording the differentiated severity of a mental illness [20]. The GAF records the general level of functioning taking into account psychological, social and professional aspects [18]. The degree of severity of functional impairment is assessed on a scale of 1-100, with a value of 100-91 reflecting excellent performance and a value of 10-1 reflecting very severely impaired performance. The duration of the illness was taken from the medical records or from information provided by the treating physician. Further inclusion criteria were: (IV) age 18 to 65 years, (V) able to give informed consent, (VI) German language proficiency sufficient to understand questionnaires and questions asked.

### Participants

**Fig. 1:**
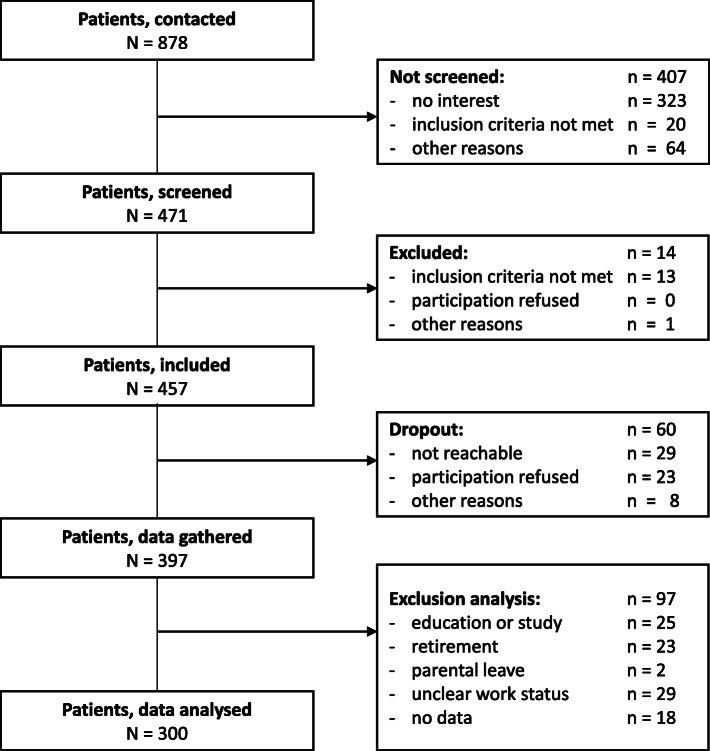
Flow chart of included patients

A total of 878 individuals were initially contacted to participate in the study. Of these, 471 were interested in participating and were screened. 457 individuals met the inclusion criteria and agreed to participate. Data were collected from 397 individuals. Data could not be gathered from 60 individuals since they were no longer reachable, later decided not to participate, or had other reasons for discontinuation. Individuals who were students (*n* = 25), retired for reasons of age (*n* = 23) or taking parental leave (*n* = 2) were not included in the analysis because they were considered to be unavailable to participate in the labour market. For 29 individuals, the information on employment status was unclear and for another 18 individuals, no data on their work status were available. Thus, analyses were carried out using the data collected from 300 individuals (Fig. 1). For nine of the included participants, only the fulfilment of the inclusion criteria was documented, but concrete values are missing for GAF (*n* = 7) or age (*n* = 2).

### Variables

#### Outcome variable

Information on employment status stems from the questionnaire "Client Sociodemographic and Service Receipt Inventory" (CSSRI) [21]. The CSSRI is a semi-structured interview to collect social and demographic data, data on employment, accommodation, detailed information on treatment, physician visits and the use of social and health services to estimate health costs. The interview includes the question: “Do you currently have a job (this includes unpaid and sheltered work)? If not, are you currently undergoing training or drawing a pension?” Following this, the information was classified into different categories: (1) competitive employment, (2) no competitive employment (including sheltered work activity, unemployed, disability pension, minor employment^1^), (3) other (including education/study, retirement, parental leave and unclear work status). The data of participants from category 3 were not considered for the current analyses. For the analyses, a binary variable of employment status was formed, distinguishing individuals with a competitive employment (i.e., 1 “yes”) from individuals without a competitive employment (i.e., 0 “no”).

#### Determinants

The following data were collected: socio-demographic information (age, sex, education, marital status, living situation, migration background), medical history (psychosocial functioning, age at onset of mental problem, presence of a chronic physical illness) and employment related data. If respondents or one of their parents were born abroad, they were considered to have a migration background. Furthermore, individuals living alone were distinguished from those not currently living alone (with a partner, children, parents, siblings, other relatives, with friends or others). The self-rated current ability to work compared to the best ability to work ever achieved was surveyed using the Work Ability Index 1 (WAI 1) [22]. WAI 1 consists of a single item, “Assume that your work ability at its best has a value of 10 points. How many points would you give your current work ability?” (0 = Completely unable to work, 10 = Work ability at its best). A score of 10 corresponds to an excellent subjective ability to work, 8 to 9 to a good, 6 to 7 to a moderate and a score of 0 to 5 to a poor subjective ability to work [23].

### Analyses

Absolute and relative frequencies as well as means and SDs were calculated as descriptive statistics. Group comparisons between the ‘competitive employment’ group and the ‘no competitive employment’ group were calculated using Pearson’s Chi-squared test for categorical variables and Two-Sample Wilcoxon rank-sum (Mann-Whitney) tests for continuous variables. The relationship of determinants with competitive employment was analysed using a hierarchical binary logistic regression model, examining the relationship of socio-demographic, health-related and individual work-related factors with the likelihood of having a competitive employment. A two-sided *p* < 0.05 was considered statistically significant. Wald χ^2^ statistics were used to test the significance of predictor variables in the model. Data were missing for <10 % of all covariates and handled by case-wise deletion, since sensitivity analysis revealed no indication of systematic biases due to missing data. The corresponding sample sizes of each subgroup comparison are reported in the tables. All statistical analyses were performed using Stata 15.1 MP (StataCorp LP, College Station, TX) and IBM SPSS Statistics for Windows, version 24 (IBM Corp., Armonk. NY).

## Results

### Sociodemographic, illness-related and work-related characteristics

In total, 300 individuals with a mean age of 42.8 years (SD 12.4) were included in the analysis. More than half of the participants were women (*n* = 167, 55.7 %), or single (*n* = 178, 59.3 %). 128 participants (43.4 %) lived alone. 50 participants (16.7 %) reported that they were (or at least one parent was) born in another country. The mean psychosocial impairment of participants was considerable (GAF, mean (SD): 42.5 (10.0)) corresponding to severe disease impairment [24]. More than half of the participants reported comorbid physical diseases (*n* = 159, 53.2 %). Work ability (WAI 1) was given an average rating of 4.0 (SD: 2.8). Further characteristics of the study participants are shown in Table 1.

**Table 1:** Sociodemographic, clinical and other characteristics of study participants according to their work status

	All participants	Competitive employment		Value Wilcoxon Two-Sample test/ Chi-squared test (Pearson) (df)
	***N*** = 300	No ***n*** = 197 (65.7)	Yes ***n*** = 103 (34.3)	
**Age (years), mean (SD)** (*n* = 298)	42.8 (12.4)	42.7 (12.7)	42.9 (12.0)	z = -0.047
**Sex, n (%)**				
Male	133 (44.3)	81 (41.1)	52 (50.5)	χ^2^ = 2.40 (1)
Female	167 (55.7)	116 (58.9)	51 (49.5)	
**Education, n (%)** (*n* = 299)				
Low	125 (41.8)	87 (44.4)	38 (36.9)	χ^2^ = 2.81 (2)
Medium	87 (29.1)	58 (29.6)	29 (28.2)	
High	87 (29.1)	51 (26.0)	36 (34.9)	
**Marital status, n (%)**				
Single	178 (59.3)	122 (61.9)	56 (54.4)	**χ**^**2**^ = 13.09 (2)**
Married/registered partnership	60 (20.0)	28 (14.2)	32 (31.0)	
Divorced/widowed/separated	62 (20.7)	47 (23.9)	15 (14.6)	
**Living situation, n (%)** (*n* = 295)				
Alone	128 (43.4)	88 (45.6)	40 (39.2)	χ^2^ = 1.11 (1)
Not alone	167 (56.6)	105 (54.4)	62 (60.8)	
**Migration background, n (%)**				
No	250 (83.3)	162 (82.2)	88 (85.4)	χ^2^ = 0.50 (1)
Yes	50 (16.7)	35 (17.8)	15 (14.6)	
**GAF, means (SD)** (*n* = 293)	42.5 (10.0)	40.1 (9.4)	46.9 (9.6)	z = -5.40 ***
**Age at first mental problems**				
**(years), mean (SD)** (*n* = 280)	25.9 (12.3)	24.9 (11.9)	27.7 (12.9)	Z = -1.62
**Physical illness, n (%)** (*n* = 299)				
No	140 (46.8)	79 (40.3)	61 (59.2)	**χ**^**2**^ = 9.70 (1)**
Yes	159 (53.2)	117 (59.7)	42 (40.8)	
**Work ability, mean (SD)** (*n* = 293)	4.0 (2.8)	3.7 (2.8)	4.7 (2.7)	z = -2.84 *

Notes: Subsample sizes vary due to missing information. Corresponding sizes for variables with missing data are given in brackets. *GAF* Global assessment of functioning, *SD* standard deviation, *n* number of participants, *df* degrees of freedom

Boldface indicates statistical significance: *p<0.05, **p<0.01, ***p<0.001

**Fig. 2:**
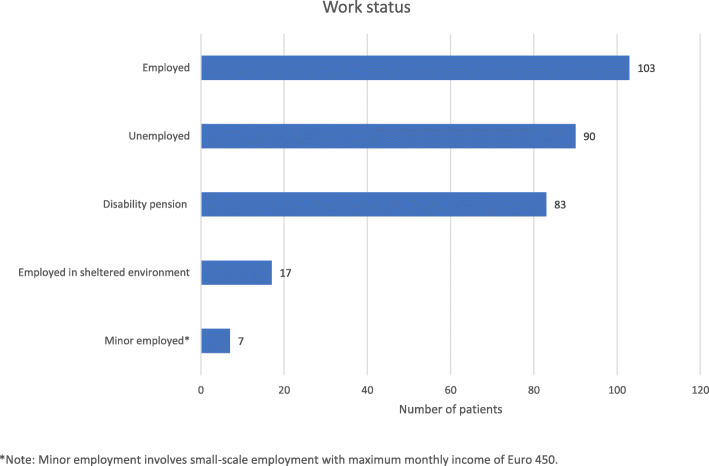
Work status of included patients

Of the group of 300 individuals included in the analysis, 103 (34.3 %) were competitively employed and 197 (65.7 %) of the individuals surveyed were not (Table 1). Of the 197 participants, 17 (5.7 %) were in sheltered employment, 83 (27.7 %) received a disability pension, and 90 (30.0 %) were unemployed. 7 (2.3%) participants reported minor employment (Fig. 2). It must be taken into account that respondents who were students either in academic or vocational training, retired for reasons of age or on parental leave were not included in the analysis (Fig. 1).

### Predictors of competitive employment

Table 1 gives an overview of the differences between the participants with competitive employment compared to those without competitive employment. With regard to sociodemographic variables, the results show that having a job and marital status are related (Pearson Chi2(2) = 13.08, *p* = 0.001). Participants with competitive employment are more often married and, in a partnership, and less often divorced, separated, or widowed. No differences were observed with regard to age, gender, education, living situation and migration. In terms of illness, there was a difference, with greater psychosocial impairment (mean GAF 40.1 vs. 46.9, Wilcoxon rank-sum test: z = -5.40, *p* < 0.001) and a higher proportion of additional chronic physical illnesses (59.7 % vs. 40.8 %, Pearson Chi2(1) = 9.70, *p* = 0.002) seen in the group of individuals not competitively employed. The age at which mental health problems first occurred did not differ significantly between the two groups. Findings also point to a difference in current ability to work. This was rated as significantly better by participants with competitive employment (WAI 1: Mean 4.7 vs. 3.7, Wilcoxon rank-sum test: z = -2.84, *p* < 0.004).

Results of the binary logistic regression analysis (*n* = 262 cases with complete data) are shown in Table 2. The model indicated that chronic physical illness was negatively associated with competitive employment, adjusting for sociodemographic and other variables. Having a comorbid chronic physical illness is 2.6 times more likely to be without a competitive employment compared to being in a status without a chronic physical illness (OR = 0.38 [95% CI: 0.21 – 0.71], *p* = 0.002). The model also showed an association of competitive employment with psychosocial functioning. For a one-unit increase on the GAF scale, the odds of being in a competitive employment increased by 9% (OR = 1.09 [95% CI 1.05 – 1.13], *p* < 0.001). All sociodemographic variables examined in the model, including marital status, had no significant effect on competitive employment. The influence of subjective work ability was also no longer significant when other variables were included. The model explained 18% of variance (*R*^2^ = .178).

**Table 2:** Social, health and work related determinants for competitive employment in individuals with SMI

Indicator variable	Category	Model (*n* = 262)		
		OR	95% CI	
**Gender**	Female	Ref.		
	Male	1.26	0.70	2.26
**Age**		0.99	0.96	1.03
**Education**	Low	Ref.	*χ*^*2*^ *=0.121*	
	Medium	0.74	0.37	1.51
	High	1.47	0.72	2.99
**Marital status**	Single	Ref.	*χ*^*2*^ *=0.127*	
	Married	2.07	0.84	5.11
	Divorced/ widowed/ separated	0.71	0.30	1.67
**Living situation**	Alone	Ref.		
	Not alone	0.76	0.38	1.53
**Migration background**	No	Ref.		
	Yes	0.70	0.30	1.64
**Age at first problems**		1.02	0.99	1.05
**GAF**		1.09***	1.05	1.13
**Physical illness**	No	Ref.		
	Yes	0.38**	0.21	0.71
**Work ability**		1.04	0.94	1.16
	*R*^*2*^ *(McFadden)*	*0.178*		

Notes: *GAF* Global assessment of functioning, *OR* odds ratio, *CI* confidence interval, Boldface indicates statistical significance: *p<0.05, **p<0.01, ***p<0.001, χ^2^ indicating the significance of the predictor variable in the models by Wald test

## Discussion

### Employment status

Our data confirm labour market exclusion of many individuals with severe mental illness (SMI). At the same time, a substantial proportion of respondents (34%) was competitively employed. This is an important resource for the individuals concerned, as employment is associated with numerous positive effects. The work rate that we report is somewhat higher than reported in a similar survey [14]. The authors reported that 21% of surveyed individuals in inpatient treatment had a fixed employment contract. However, differences were identified based on treatment setting (day clinic, open vs. closed wards) and diagnoses. A recent study, also conducted in a clinical setting, found that 34% of individuals with mental illness worked competitively or were engaged as students in vocational training or academic studies [25]. It must be assumed that variations in employment rates are also dependent on sampling and the definition of inclusion criteria. In the present study individuals with (SMI) were examined. The operationalization was based on the work of Ruggeri et al. [26]. Inclusion was limited to individuals with a diagnosis of schizophrenia and severe affective disorder. In the study by Jäckel et al. (2020) a survey of all individuals in day hospital and inpatient psychiatric treatment with informed consent was conducted. In addition, vocational training and employment were recorded simultaneously here [25]. In the study by Mernyi et al. (2020), a screening of all individuals in inpatient psychiatric treatment was conducted [14]. Internationally, the work rates of individuals with SMI are also estimated to be at a low level of 10 to 15% (see [27]).

### Predictors of work

Our results further indicated that psychosocial functioning and chronic physical illness could have an influence on the working status. The **influence of psychosocial functioning** and mental illness on work is in line with other studies [28–30]. In a 5-year prospective, longitudinal study based on a sample of 529 individuals with SMI who met criteria for sustained competitive employment at study entry, more than half of the participants experienced work interruptions during the follow-up period. Results revealed that participants who reported fewer problems with daily functioning were more likely to work a greater number of months over the course of the 5-year study [29]. Kortrijk et al. (2019) measured the proportions of 2150 individuals with SMI treated in Flexible Assertive Community Treatment teams who were unemployed and gained employment and who were employed and lost to employment. Over time, 10% remained employed, 5% lost their employment, 3% gained employment and 82% remained unemployed. Individuals who found employment were younger, more often male, and had significantly fewer psychosocial problems during follow-up than those who remained unemployed [28]. Psychosocial function appears to play a role in both finding a job and in job retention.

SMI is defined in part by impaired psychosocial functioning [26]. Improvement of functioning is achieved through the use of various psychosocial interventions aimed at strengthening abilities, such as cognitive or social skills [31]; such interventions include social skills training [32, 33], cognitive remediation [34] and occupational therapy [35]. In the field of rehabilitation, various training courses have been developed that specifically train critical work-related skills [36–38]. Effectiveness studies have shown that Supported Employment (SE) augmented with other specific interventions is an effective tool for people with SMI in terms of finding and retaining jobs [39]. SE is considered as an evidence-based strategy for promoting employment in individuals with SMI [40]. The SE model most clearly described and most frequently evaluated is Individual Placement and Support (IPS). Core IPS principles include rapid placement in competitive employment, attention to individuals preferences, systematic job development, integration of mental health and employment services, and support from specialized services [41]. Several reviews and meta-analyses have demonstrated the superiority of IPS over traditional vocational rehabilitation approaches, such as pre-vocational training or transitional employment, in terms of obtaining and maintaining employment of individuals with SMI [39, 42, 43] across a variety of settings and economic conditions [40]. Individuals who receive IPS are more than twice as likely to gain competitive employment compared to participants who receive other interventions [39, 40, 42]. Despite the convincing evidence in favour of the IPS model, it must be taken into account that a considerable proportion of program participants do not find a job. Several studies have therefore investigated the effects of combined strategies on employment attainment and retention. Augmented SE, supplemented, for example, by specialized work-related social skills training [37, 44] or cognitive skills training [45], may be somewhat more effective compared to SE alone in obtaining competitive employment [39]. There is a lack of studies on the impact on non-vocational outcomes. However, findings indicate that training measures need to be tailored to individual needs. For example, SE augmented with cognitive enhancement was shown to improve occupational outcomes for participants with low community functioning. There were no effects for participants with higher levels of psychosocial functioning [46].

The **presence of a comorbid chronic physical illness** (vs no physical illness) reduced the odds ratio of respondents having a competitive employment by more than 60%. A previous study of SE in unemployed individuals showed that having a physical comorbid condition was predictive of lower rates of competitive employment, fewer hours worked, and lower wages earned over the 2-year follow-up period [47]. This finding has high relevance for the treatment and rehabilitation of individuals with SMI. It is known that the risks of somatic morbidity are comparatively high in individuals with SMI. This is particularly the case for cardiovascular disease, chronic obstructive pulmonary disease, diabetes, and infections [48–50]. The causes are manifold, and they include, e.g., unhealthy lifestyles, adverse effects of drugs or reduced access to somatic care [51, 52]. For example, many people with SMI seem to have insufficient contact with their GP because of their somatic health problems [53]. A lack of physical health care for this population group is associated with various factors. On a personal level, the use of physical health services may be affected by the exacerbation of psychiatric symptoms. Structural barriers also play a role. These include, in particular, a lack of continuous health monitoring and of psychosocial interventions [54]. Increasing attention is given to interventions aimed at promoting healthy lifestyles and improving physical health care among individuals with SMI [31]. In general, multimodal interventions have led to weight reduction and positive metabolic outcomes [55–57]. Other approaches have also been developed to improve physical health in Individuals with SMI, such as promoting smoking cessation, physical health monitoring and changes in healthcare organization [58, 59]. The latter is primarily aimed at the coordination of somatic health care in individuals with SMI [60]. Such integrated models of primary medical care for individuals with SMI increase the likelihood of GP treatment and use of preventive measures [60]. A more integrated approach appears to have positive effects on health outcomes, patient satisfaction and health-related quality of life; the use of peer-interventions could improve health service utilization [61, 62]. The positive predictive power of physical health status for job retention underscores the role of integrated care for individuals with SMI including mental health, psychosocial and somatic care measures.

Previous studies of predictors of employment have shown that subjectively perceived impaired work ability is a significant barrier to individuals finding jobs [63, 64]. Our analyses showed that subjective work ability lost its predictive power when various sociodemographic and health-related factors were taken into account. It is conceivable that the effect is masked by physical illness and psychosocial functioning. In addition to intrapersonal (physical and psychological) skills environment-related support factors or barriers have also been discussed [65–68]. These were not considered here. Workplace characteristics may have an impact on employment retention and require further study. Evidence can be found, for example, on the importance of workplace social networks [69], job matching [70], job satisfaction [71], time demands [72, 73] and supervisor interaction [74]. These factors were not taken into account in this study, which reduces the strength of the results.

### Limitations

The investigation is subject to limitations that must be taken into account when interpreting the results. Although a large sample of individuals with SMI was investigated, selection bias cannot be ruled out. This holds true with respect to recruitment strategies and to the response to study invitation. The results refer only to individuals with SMI in the Bavarian region, Germany. Participants without competitive employment are slightly underrepresented in the analysis of covariate missing’s. In the multivariate model, however, there is no unacceptable selection bias due to case-wise deletion. Furthermore, a large part of the data is based on self-report by study participants. This applies to employment status, but also to somatic comorbidity, subjective work ability and socio-demographic data. The data were collected in a cross-sectional study so that co-variations cannot be interpreted causally. The level of explained variance in our model indicates that other variables also play a role.

## Conclusions

The findings show that one-third of individuals with SMI is competitively employed and almost one-third is unemployed. A substantial subgroup has retired early. Almost 60% of those affected are thus excluded from employment. Lower levels of psychosocial functional impairment and the absence of additional chronic physical illness have been shown to contribute substantially to individuals holding jobs. The increased risk of somatic comorbidity in individuals with SMI and its unfavourable consequences, not only in terms of poorer participation in work, justify a high priority for integrated care for this group of individuals with close integration of medical, psychiatric and rehabilitative services. Only integrated care and consideration of the person as a whole will meet the complex needs of individuals with severe mental illness.

## Data Availability

The datasets used and analysed in the presented analyses are available from the corresponding author upon reasonable request.

## Abbreviations

CI: Confidence interval
CSSRI: Client Sociodemographic and Service Receipt Inventory
df: Degrees of freedom
DRKS: German Clinical Trials Register
GAF: Global Assessment of Functioning
HoNOS: Health of the Nation Outcome Scales
ICD: International Statistical Classification of Diseases and Related Health Problems
IMPPETUS: Implementation of the patient guideline Psychosocial therapies for Patients with Severe Mental Illness
M: Means
n: Number of participants
OR: Odds ratio
p: *P*-value
SD: Standard deviations
T: T-statistics
SMI: Severe mental illness
WAI: Work Ability Index
X^2^: Chi^2^-statistics

## Footnotes

1 Minor employment involves small-scale employment with maximum monthly income of Euro 450.
